# Effect of Zinc Supplementation on the Frequency and Consistency of Stool in Children with Acute Diarrhea

**DOI:** 10.7759/cureus.4217

**Published:** 2019-03-11

**Authors:** Ghulam Shabbir Laghari, Zahid Hussain, Huma Shahzad

**Affiliations:** 1 Pediatrics, Liaquat University of Medical and Health Sciences, Jamshoro, PAK; 2 Pediatrics, National Institute of Child Health, Karachi, PAK; 3 Internal Medicine, Dow University of Health Sciences, Karachi, PAK

**Keywords:** acute diarrhea, oral zinc supplementation, pakistan, pediatric diarrhea, zinc in diarrhea

## Abstract

Introduction

Acute diarrhea in young children is a prevalent and distressing pediatric illness. The role of zinc therapy in the improvement of stool consistency and the shortening of the duration of diarrhea is still controversial. The aim of this study is to assess the effect of oral zinc supplementation in acute diarrhea.

Methods

Children of age 28 days till five years presenting in the outpatient department with acute diarrhea were included. Oral zinc supplementation was included in the anti-diarrheal regime of half of the children (n=50); the other half (n=50) were not given zinc. Mean body weight and the frequency and consistency of stool were noted for both groups on Days 1 and 3.

Results

The zinc group showed a significantly reduced frequency of diarrheal episodes on the third day of intervention (p<.00001). More children in the zinc group had soft to firm stool consistency than in the non-zinc group (p=.01).

Conclusion

Oral zinc supplementation has a promising role in reducing the duration of diarrhea and improving stool consistency in children with acute diarrhea. Oral zinc supplementation should be made a mandatory part of the anti-diarrheal regime for Pakistani children.

## Introduction

Despite the promising role of oral rehydration solution (ORS), acute diarrhea is one of the major preventable causes of childhood deaths. Globally, diarrhea is still the most common pediatric morbidity, with 1.5 billion episodes and almost two million deaths per year [1-2]. In middle-to-low income countries, the prevailing scenario is even worse. According to the Pakistan Demographic and Health Survey conducted in 2013, almost 23% of children age five years and under had suffered from diarrhea two weeks preceding the survey. The prevalence of diarrhea was highest among children aged six and eleven months (35%); a span during which solid foods are first introduced into the child’s diet [3].

The World Health Organization (WHO) has recommended zinc supplementation for 10 to 14 days along with low osmolarity oral rehydration solution (ORS) in acute diarrheal episode [4]. The role of oral zinc supplementation in acute pediatric diarrhea has been related to its ability to modify the host's resistance mechanism toward the infectious agents, thereby, reducing the risk, frequency, and duration of diarrhea. Zinc also plays a critical role in modulating the cell membrane and cellular function, thereby, improving immunity [5]. A study conducted by Trivedia et al., in 2013, showed a 62% reduction in stool frequency per day in the zinc group as compared to only 26% in the placebo-supplemented group [6]. In a systemic review published in 2010, it was concluded that oral zinc supplementation shortens the mean duration of acute diarrhea by up to 20% and persistent diarrhea by up to 30% [7].

Despite the fact that Pakistan is among the first countries to include zinc in its pediatric diarrhea treatment protocol, only 2% of Pakistani children under the age of five with diarrhea receive zinc as part of their acute diarrhea treatment [3]. One of the core reasons is a lack of awareness regarding the role of oral zinc in pediatric diarrhea. Another important reason is the paucity of local data enforcing the role of zinc in alleviating acute pediatric diarrhea. The aim of this study is to establish substantial evidence regarding the role of zinc in improving the consistency and frequency of stool in acute diarrhea.

## Materials and methods

This was a cross-sectional, interventional study conducted in the outpatient unit of the department of pediatrics, Civil Hospital, Jamshoro, in October 2018. One hundred children, of age 28 days till five years, presenting with acute diarrhea, were recruited after the informed consent of their parents/guardians. Children with diarrhea (>14 days), dysentery (blood in stool), and with severe dehydration requiring hospital administration were not included in the study.

All children were prescribed a probiotic and low osmolarity ORS. A banana-yogurt diet was added for children who were taking solids. The sample was then divided into two groups. The first group (n=50) was given zinc supplementation additional to the above-mentioned regime; no additional zinc supplementation was added for the second group (n=50). For infants less than six months of age, 10 mg/day zinc was prescribed, and for the older age group, 20 mg/day zinc was prescribed [4]. Age, gender, history of a previous episode of diarrhea in one month, the formula of milk (breast milk vs. formula), and the duration of diarrhea were noted for all children. On Day 1, body weight, the number of diarrhea episodes, and stool consistency were noted. All three readings were repeated on Day 3 for both the groups.

Data were entered and analyzed using IBM Statistical Package for the Social Sciences (SPSS) Statistics for Windows, version 21.0. (IBM Corp., Armonk, NY, US). Frequency and percentages were calculated for categorical data. Mean and standard deviation (SD) were calculated for continuous data. The readings of both treatment groups on Days 1 and 3 were compared via the chi-square test. A p-value of ≤0.05 was taken as significant.

## Results

There were 50 children in each group. The zinc group had more male children (58%) and the non-zinc group had more female children (52%). The overall mean age of the sample was 25.12±6.05 months. The characteristics of the children, including the mean age, gender, history of acute diarrhea episodes in the last month, form of milk intake, duration of current diarrhea episode, and number of loose stools per day for both study groups, are shown in Table 1.

**Table 1:** Characteristics of the patient in the zinc and non-zinc groups (n=50)

Patient characteristic	Zinc group n (%)	Non-zinc group n (%)
Gender		
Male	29 (58%)	24 (48%)
Female	21 (42%)	26 (52%)
Previous history of acute diarrhea within one-month	13 (26%)	28 (56%)
Milk formula		
Breastfeeding	6 (12%)	7 (14%)
Formula milk (liquid)	19 (38%)	14 (28%)
Formula milk (powdered)	13 (26%)	18 (36%)
No milk intake	12 (24%)	11 (2%)
Age in months (mean ± SD)	24.63 ± 3.58	29.38 ± 5.74
Duration of diarrhea in days (mean ± SD)	3.05 ± 1.24	4.85 ± 1.48
Frequency of loose stools / day (mean ± SD)	2.57 ± 1.11	3.25 ± 2.04

The body weight, frequency of diarrheal episodes per day and the stool consistency for both groups were compared on Days 1 and 3. As seen in Table 2, the mean body weight and mean frequency of diarrheal episodes on Day 1 were comparable for both study groups. In the zinc group, 56% of children had stools of watery consistency. In the non-zinc group, there were 72% of children with watery diarrhea. However, on Day 3, children in the non-zinc group were noticed to have a significantly lower mean body weight as compared to the zinc group. The mean frequency of diarrheal episodes was significantly less in the zinc group than the non-zinc group. As far as the stool consistency was concerned, the zinc group showed more improvement (p-value = 0.01). From 56% of children with watery diarrhea on Day 1, there were only 14% of children with watery diarrhea on Day 3 in the zinc group. There were no children with firm stools in the zinc group on Day 1 and by Day 3, 54% of children had firm stools. On the other hand, in the non-zinc group, there were 72% children with watery diarrhea on Day 1, which only reduced to 38% on Day 3 (as compared to 14% in the zinc group). There were no children with firm stools in the non-zinc group on Day 1 and by Day 3, only 32% children had firm stools (as compared to 54% in the zinc group) (Table 2).

**Table 2:** Comparison of the frequency and consistency of stool in the zinc and non-zinc groups on Days 1 and 3 of supplementation

Variable	Zinc group	Non-zinc group	P value
Day 1:			
Mean body weight (kg)	12.58 ± 2.06	11.89 ± 2.65	0.14
Mean frequency of diarrheal episodes	6.14 ± 0.98	6.37 ± 1.04	0.25
Stool consistency (%)			0.09
Watery	28 (56%)	36 (72%)	
Soft	22 (44%)	14 (28%)	
Firm	--	--	
Day 3:			
Mean body weight	11.06 ± 1.04	9.05 ± 1.85	< 0.00001
Mean frequency of diarrheal episodes	2.40 ± 0.81	4.28 ± 1.07	< 0.00001
Stool consistency			0.01
Watery	7 (14%)	19 (38%)	
Soft	16 (32%)	15 (30%)	
Firm	27 (54%)	16 (32%)	

## Discussion

Zinc supplementation for acute diarrhea in young children was first recommended by WHO in 2004 [4]. Although Pakistan is among the first countries to include zinc in its diarrhea management, still according to Pakistan Health Survey (2012), only 2% of children were receiving zinc for diarrhea, which only increased to 8% in the 2017-2018 survey [3,8]. Furthermore, according to the National Nutrition Survey 2011, 39.2% of Pakistani children of ages zero to five years were zinc deficient [9]. Our study concluded a significantly improved stool consistency and a reduced frequency of daily episodes of diarrhea with oral zinc supplementation.

In the past, there have been contradictory studies regarding the role of oral zinc supplementation in acute pediatric diarrhea. In a randomized open-label study from India, adding oral zinc supplementation to standard anti-diarrhea therapy resulted in less time to resolution [10]. However, a study with Polish children failed to show any significant impact of adding zinc to the anti-diarrhea regime in their pediatric population. They attribute their results to the fact that their children are not zinc-deficient [11]. This hypothesis that zinc supplementation will only benefit children with zinc deficiency is also enforced by another multinational study. Walker et al. conducted a study with infants of age 28 days till five months in Pakistan, India, and Ethiopia. The study did not find any difference in the stool frequency and rates of vomiting in the zinc and placebo groups. They also attributed their results to the fact that exclusively and predominantly breastfed infants already have a higher zinc intake than older children taking solids [12].

However, evidence supporting the role of zinc in acute pediatric diarrhea is still strong. Oral zinc supplementation significantly improved the duration of diarrhea and reduced the volume and frequency of stools within 24 hours of initiation of therapy [13]. Children being supplemented with oral zinc have better response rates, shorter duration of diarrhea, and a significantly lower recurrence rate within three months [14]. In another study, only 5% of children of the zinc group had diarrhea after 120 hours of intervention as compared to 20% of the placebo group. Furthermore, the zinc group had a lower frequency of diarrhea [15].

Diarrhea is among the most common and most prevalent pediatric illnesses, especially in middle-to-low income countries. Scientific evidence regarding the role of zinc supplementation in acute pediatric diarrhea is controversial. Large-scale, double-blind trials must be conducted to bring substantial evidence to the table. Longitudinal studies to understand the preventive role of zinc in reducing the recurrence of diarrhea in children are also the need of the hour. Pediatric infectious and public health specialists must take aggressive steps to ensure the inclusion of zinc supplementation in the anti-diarrhea regime for all Pakistani children.

## Conclusions

Oral zinc supplementation has a promising role in reducing the duration of diarrhea and improving stool consistency in children with acute diarrhea. Substantial research is still needed in this sector to reinforce the role of zinc in not only alleviating current diarrheal episodes but also in the prevention of diarrheal recurrence. Oral zinc supplementation should be made a mandatory part of the anti-diarrheal regime for Pakistani children.
